# Medical Appointments

**Published:** 1849-06

**Authors:** 


*Medical Appointments.*—Prof. Bartlett has been appointed to, and accepted, the chair of medicine in the Louisville medical school, vacated by the resignation of Prof. Drake. Prof. Drake, it is understood, will become a member of the Faculty of the Ohio medical school, Cincinnati.

Dr. Hunt, of New Orleans, has been elected to fill the vacancy in the medical department of the University of Louisiana, occasioned by the death of Dr. Harrison. Dr. Benjamin Silliman, jr., has been appointed to the chair of chemistry in the University of Louisville, and Prof. Yandell, formerly the incumbent of that chair, transferred to the chair of Physiology and Pathological anatomy.

Dr. H. J. Bigelow, of Boston, has been elected to the chair of clinical surgery in the medical department of Harvard University; Prof. Geo. Hayward having resigned. Dr. Bigelow is a son of Dr. Jacob Bigelow, Professor of materia medica, in the same school.

Dr. F. Campbell Stewart, of the city of New York, has been appointed Physician of the Marine Hospital, N. Y.

